# Decoding the Role of Gender in the Relationship Between the Online Payment System and SME Performance: A Case Study Investigating an Emerging Economy—Bangladesh

**DOI:** 10.3389/frma.2022.842670

**Published:** 2022-03-22

**Authors:** Sharmin Nahar

**Affiliations:** Birmingham City Business School, Birmingham City University, Birmingham, United Kingdom

**Keywords:** online payment system (OPS), business performance, SMEs, gender, technology acceptance model (TAM), resource-based view (RBV), emerging economies, Bangladesh

## Abstract

This research examines the moderating role of gender on the adoption of online payment systems (OPS). It also explores the impact of using OPS on the performance of SMEs in an emerging economy, using the Technology Acceptance Model (TAM) and Resource-Based View (RBV). The results indicate that male SME owners (entrepreneurs) are more likely to focus on perceived usefulness, whereas female SME entrepreneurs are more likely to focus on perceived ease of use while adopting OPS, according to data obtained from 302 SMEs in Bangladesh through face-to-face surveys. The results also report that the use of OPS has a considerable positive effect on SMEs' performance. The study's findings will add to the scarce research on the impact of using OPS on business performance in the context of SMEs in emerging economies, in addition to enhancing the OPS adoption literature from a gendered lens.

## Introduction

In the last few years, the rapid diffusion of the internet has played a major role in individual and business practises (Greenstein, 2020). Especially, the use of the internet on the mobile platform has made it easy for a considerable number of people to access the internet. Mobile internet is accessed by at least 5.9 billion people of around 6.5 billion total global subscribers of mobile phones-making it one of the most widely utilised information and communication technologies (ICT) in daily life (Ericsson, 2021). An example of such an innovative service that has a global impact on business practises is the online payment system. This influence, on the other hand, has not been thoroughly examined in academia. While the existing literature suggests that mobile telephony usage and dissemination have increased over time (Zhu et al., 2021), there is little evidence of exploring innovative mobile services like online payment systems (OPS) in the context of emerging economies. With the introduction of e-commerce and the rise of the Internet, the payment process became more digitised, with the availability of numerous online payment methods such as electronic cash, debit cards, credit cards, contactless payment, mobile wallets, and so on. Furthermore, mobile payment services are growing in popularity day by day and are demonstrating a change by moving toward a promising future of opportunities (Rouibah et al., 2016; Khan et al., 2017; Nguyen and Nguyen, 2020).

Furthermore, there has been little research on the adoption of OPS in Small and Medium Enterprises (SMEs), let alone the implications of such adoption. Using this as a backdrop, this study examines the adoption of OPS and its impact on company performance in Bangladeshi SMEs.

Majority of the existing literature focuses on the factors that impact the adoption of OPS (e.g., He and Mykytyn, 2007; Khan et al., 2017; Shankar and Datta, 2018; Vally and Divya, 2018; de Luna et al., 2019; Park C. et al., 2019; Park J. et al., 2019; Al-Saedi et al., 2020). However, there exist very few studies concentrating on the impact of OPS on firm performance exclusively. This is more so non-existent in the context of Bangladesh despite the recent boom in OPS adoption in the country (The Daily Star, 2021), which is the fastest-growing economy in the Asia-Pacific region (Ziauddin, 2019). I especially concentrated on SMEs since financial inclusion or access to the OPS is still a bigger bottleneck for SMEs compared to larger firms (Rajan, 2016; Baer et al., 2018; Kramon and Weghorst, 2019).

I also explored how the gender of the entrepreneurs/owner-managers impact the relationship between OPS use and SME performance relationship. This is also unexplored in the existing literature, yet important to explore since prior research reports differences in the way male and female users interact with ICT (Guadagno et al., 2011), and men, in general, have a more positive outlook toward ICT compared to women (Kesici et al., 2009; Hasan, 2010). This gender difference in technology use is even more pronounced in a country like Bangladesh, where both the genders are conditioned differently, and it has a resultant impact on the way they approach technology.

So, this study explores two central research questions. First, does the gender of the entrepreneur/owner-manager influence the adoption of online payment systems (OPS) in emerging economies? Second, does the use of OPS impact SME performance in emerging economies?

The remainder of the paper is organised as follows. In the section “Literature Review and Research Framework,” I review existing literature to aggregate different independent, dependent, and moderator variables used in different papers to build my research model. The research strategy, data collection, measurements, and analysis method used for the study are explained in the section “Research Methodology.” The results are presented in the section “Results.” My research findings, implications for future research, and limitations of my study are provided in the section “Discussion and Conclusion.”

## Literature Review and Research Framework

### Technology Acceptance Model (TAM)

The TAM's central idea is that a person's attitude toward technology is influenced by both perceived usefulness (PU) and perceived ease of use (PEU). Technology usage is determined by a person's behavioural intention (BI) to use it, which differs from the notion of reasoned action in that it is considered as being mediated by the person's attitudes toward utilising it (Granić and Marangunić, 2019).

The TAM's attitude-behavioural intentions relationship argues that all uses are equal and that intentions to use technology again can be created based on positive technology usage. This is assumed by the PU–BI relationship, which has been shown to have a favourable or negative impact on individual behaviour in organisations (Granić and Marangunić, 2019). As a result, the study aims to assess perceptions and linkages among various TAM dominants, using gender as a moderator.

#### Perceived Ease of Use

The degree to which a person believes that utilising the system would be effortless is characterised as perceived ease of use (PEU) (Davis, 1989). To get the most out of his online purchase, the buyer must be familiar with the processes of searching for data, placing orders, and obtaining customer support (Makame et al., 2014; Alshurideh et al., 2019). Ahmad et al. (2020) found that customers who have favourable attitudes about the ease of use and utility of internet purchases are more likely to employ e-payment systems. Alshurideh et al. (2021) futher suggested that because of the additional complexity of using an electronic device to make a payment, this aspect could be impacting OPS adoption. This has also been echoed by Agarwal and Prasad (1999) and Davis (1989), who suggested that PEU is also likely to have an impact on PU and BI, either directly or indirectly, through its effect on PU (Davis et al., 1989; Agarwal and Prasad, 1999). As per the above discoveries, I suggest the following hypothesis:

**H1:** Entrepreneurs' perceived ease of use (PEU) positively influences their behavioural intention (BI) of adopting the online payment systems in their SMEs.

Men are thought to adopt and use technology more extensively than women, who are considered passive consumers; yet, the number of women customers using e-payment systems has increased dramatically recently (Khan et al., 2020). Many researchers, however, overlook the issue of gender in the context of OPS (Jusoh and Jing, 2019). Hence, there hasn't been much research on the role of gender in OPS uptake and use so far. In the case of similar technologies like mobile banking, the existing literature suggests that males were more likely to use the technology as they find them easy to use compared to women (Chawla and Joshi, 2020). This has been echoed by Abdullah and Ward (2016), who suggested that men's technology self-efficacy scores are higher than women's, according to previous studies. Furthermore, Ong and Lai (2006) discovered that men rated PEU higher in terms of new technology use. Women, on the other hand, are frequently hesitant to adopt internet platforms because they believe they are complicated. This manifests as a lack of confidence and the anxiety that comes with it (Chawla and Joshi, 2020). Easy-to-use information technology systems, according to Moon and Kim (2001), will pose less of a hazard to females. Otherwise, females are generally hesitant to adopt new technologies. As per the above discoveries, I suggest the following hypothesis:

**H3:** Gender moderates perceived ease of use (PEU) toward entrepreneurs' adoption of the online payment systems in their SMEs.

#### Perceived Usefulness

The degree to which a person believes that employing a certain technology would improve their performance is described as perceived usefulness (Manis and Choi, 2019). Male users viewed technology as more valuable than female users, according to a previous study (Schmidthuber et al., 2020). According to Park C. et al. (2019) and Park J. et al. (2019), a new technology with a higher level of perceived usefulness is one in which a user believes there is a positive user-performance relationship, which has been shown to influence people's behavioural intention to use the new technology (Verma and Sinha, 2018; Manis and Choi, 2019; Park C. et al., 2019; Park J. et al., 2019). Males are more driven by instrumental reasons than women, according to Schmidthuber et al. (2020), and men evaluate perceived usefulness to a greater extent than women when making decisions about the utility or performance-related elements of new technology.

As per the above discoveries, I suggest the following hypothesis:

**H2:** Entrepreneurs' perceived usefulness (PU) positively influences their behavioural intention (BI) of adopting the online payment systems in their SMEs.
**H4:** Gender moderates perceived usefulness (PU) toward entrepreneurs' adoption of the online payment systems in their SMEs.

#### Behavioural Intention to Use

Ong and Lai (2006) note various challenges in evaluating multidimensional elements of use such as mandated vs. voluntary, informed vs. ignorant, effective vs. ineffective, and so on. According to DeLone and McLean (2003), BI could be a viable option. The difference between BI and use is that BI is an attitude and use is an action (Ong and Lai, 2006). Men have greater experience dealing with computers and have more positive views than women, according to a large body of data (Durndell and Thomson, 1997; Whitley, 1997; Ong and Lai, 2006). Furthermore, according to Reda and Dennis (1992), the behavioural intention (BI) of entrepreneurs to adopt new technology has a beneficial impact on their usage of new technology in their SMEs.

As per the above discoveries, I suggest the following hypothesis:

**H5:** Entrepreneurs' behavioural intention (BI) of adopting the online payment systems positively influences their use of the online payment systems in their SMEs.

### The Resource-Based View (RBV)

This paper is grounded in the Resource-based View (RBV hereafter) because RBV has emerged as a very popular theory for explaining firm performance over the last three decades (Newbert, 2007). As per the RBV, firms should possess strategic resources to create competitive advantages or superior business performance. According to Barney (1991), a firm's assets should be valuable, rare, imperfectly imitable, and non-substitutable to be considered as strategic resources. Barney (1991) argued that resources are dispersed heterogeneously among firms and that some resources are not entirely imitable or substitutable (Barney, 1991). When a firm possesses a distinctive or rare set of resources, this situation is called resource heterogeneity (Peteraf, 1993). And when resources cannot be imitated by competitors; this is called imperfect imitability (Barney, 1991). Then when substitute resources are not sufficient for the execution of resources effectively or efficiently as the original resources, such original resources are interpreted as non-substitutable (Barney, 1991).

Because competitive advantages are difficult to quantify (Ketchen et al., 2007), numerous scholars have tried to link strategic resources empirically with firm success (Barney and Arikan, 2001). The underlying argument is that if strategic resources are tied to the performance of firms, then there should be a competitive advantage (Crook et al., 2008). Because competitive advantage is “largely used to explain the relative performance of competitors within a given (product) market environment” (Peteraf and Barney, 2003, p. 313), a considerable number of scholars have used the concept as synonymous with firm performance (Crook et al., 2008). According to RBV, therefore, the degree to which enterprises possess strategic resources would positively affect firm output (Crook et al., 2008).

The RBV model comprises three main constructs: firm performance, organisational resources, and capabilities. The theory's dependent construct is firm performance, while the theory's main independent construct is firm resources which include:

“all of the assets, capabilities, organisational processes, firm attributes, information knowledge, etc., controlled by a firm that enables the firm to conceive of and implement strategies that improve its efficiency and effectiveness” (Barney, 1991, p. 101).

Though the online payment system has seen considerable growth in Bangladesh (The Daily Star, 2021), its use is not yet as widespread amongst SMEs (United Nations Capital Development Fund, 2019) as among the larger firms in Bangladesh. Hence, it still can be considered as a heterogeneous resource and thus enable to create a competitive advantage for an SME. It is a valuable resource as well since it provides substantial value to the users. The access, mobility, and convenience it provides (Kim et al., 2010; Su et al., 2018), is inimitable and non-substitutable unless a better technological platform becomes mainstream which is as low cost as an online payment system. Therefore, according to RBV, the online payment system meets all the criteria to be considered a strategic resource.

As per the above discoveries, I suggest the following hypothesis:

**H6:** Entrepreneurs' use of the online payment systems as a strategic resource is positively associated with the financial performance of their organisations.

### Gender as a Moderator in the OPS Use and SME Performance Relationship

In the existing literature, the impact of gender on advanced and new technology uptake and use has gotten a lot of attention. Women are less likely than men to accept and use new and advanced ICT technologies, according to research on the subject. One cause for this may be their lack of trust in their abilities to use these advanced technologies (Li et al., 2008). Men, compared to women, are more likely to put in greater effort in the face of a hurdle without overthinking the effort or the degree of the effort, according to research. As a result, when using these technologies, males emphasise the importance of instrumentality and control. Women, on the other hand, exhibit less control and a greater focus on the process when using these devices. As a result of their perception that certain technologies are difficult to use, they are less likely to adopt and use them than men (Rotter and Portugal, 1969; Hennig and Jardim, 1977). As a result of their limited utilisation, new and advanced technologies have a smaller impact on the business performance of women-led businesses (White Baker et al., 2007).

As per the above discoveries, I suggest the following hypothesis:

**H7:** Gender of the entrepreneurs moderates the OPS use and SME performance relationship in such a way that the use of OPS adds more to performance in male-led SMEs compared to female-led SMEs.

**Figure 1:**
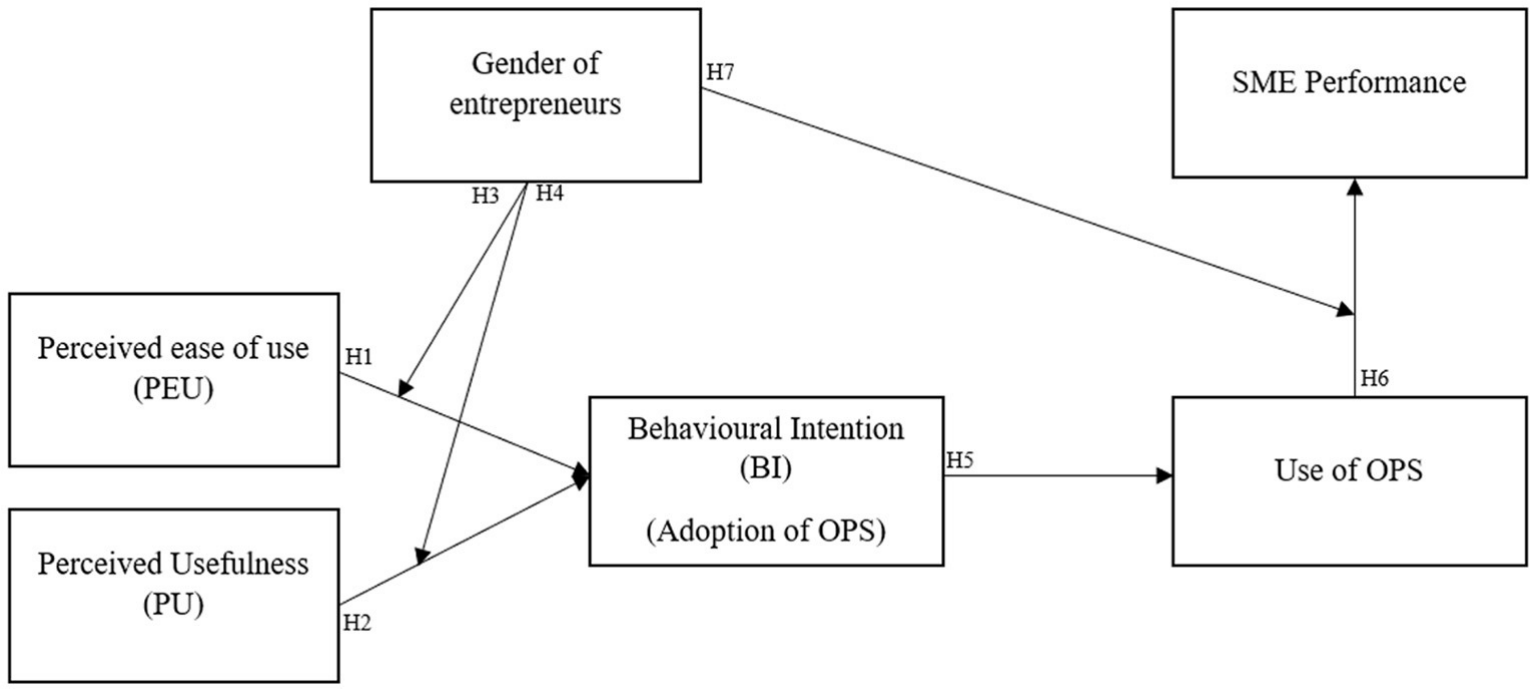
Research model.

Based on the above hypotheses, I present my research model in Figure 1.

## Research Methodology

This study incorporates data from 302 SMEs collected through face-to-face surveys with key informants, usually the founders, from two cities with significant socio-economic and infrastructural differences, based on multiple theories—Technology Acceptance Model (TAM) (Manis and Choi, 2019) and Resource-Based View (RBV) (Popli et al., 2017). If the founder is no longer associated with the company, information was gathered from the owners who own the bulk of the company. Despite the fact that SMEs are defined differently across the world, this study adheres to the previous literature (see, for example, Taylor and Banks, 1992; Cardon and Kirk, 2015) and the Bangladesh Bank's (the central bank of Bangladesh) local standard that SMEs have a maximum of 250 employees (Bangladesh Bank, 2012).

In terms of sampling technique, I randomly selected around 50% of SMEs from Dhaka city (the capital city of Bangladesh) and the other 50% from Khulna city (comparatively smaller than the capital city yet the third largest city in Bangladesh). The surveyed SMEs were chosen randomly from the databases of relevant Chambers of Commerce and Industries of two different cities of Bangladesh. These cities have major socio-economic and infrastructure contrasts, and they exhibit rural-urban dynamism, which is relevant to Bangladesh's entrepreneurial eco-system. Agriculture, retailing, crafts, readymade garments, knitwear, infrastructure, healthcare, media, etc., are among the industries represented. I also took around 50% of the samples of female-owned firms and the other 50% male-owned firms. Three hundred and two face-to-face surveys with the key informant person (founder of the SMEs) in each SME were conducted from August to October 2018.

A semi-structured questionnaire containing both closed and open-ended questions was used to collect data. In June of 2018, 350 SMEs were contacted, and the founders^1^ were invited to participate in the study. Two follow-up reminders were sent 2 and 4 weeks after the initial contact. Three hundred and twenty printed versions of the questionnaires were distributed to the respondents and after a stipulated period set by the researcher, 302 Face-to-face surveys with the key informant person (founder/owner of the SMEs) in each SME were conducted from August to October 2018, reflecting a 94.37% effective response rate. The requirement of “informed consent” was tackled by taking approval from the participants. Moreover, the four fundamental ethical standards; “no harm to participants, no lack of informed consent, no invasion of privacy and no deception” (Diener and Crandall, 1978, as cited in Bryman and Bell, 2007, p. 132) have been applied.

Industry/Sector Categorisation of the collected data is provided in Table 1.

**Table 1:** Industry/Sector categorisation.

**Sector of business**	**Freq**.	**Percent**
Agribusiness	23	7.615
Beauty salon	23	7.615
Handicrafts	23	7.615
Retailer/Grocery shop	23	7.615
Outsourcing (including content makers)	23	7.615
Textile boutique shop	23	7.615
Knitwear and readymade garments	23	7.615
ICT	23	7.615
Educational and consultancy services	23	7.615
Healthcare and pharmaceuticals	23	7.615
Infrastructure (Property, Transport and Storage)	23	7.615
Restaurant & Catering service	23	7.615
Broadcasting, advertising and event management	23	7.615
Others	3	0.99
**Total**	**302**	**100**

**Figure 2:**
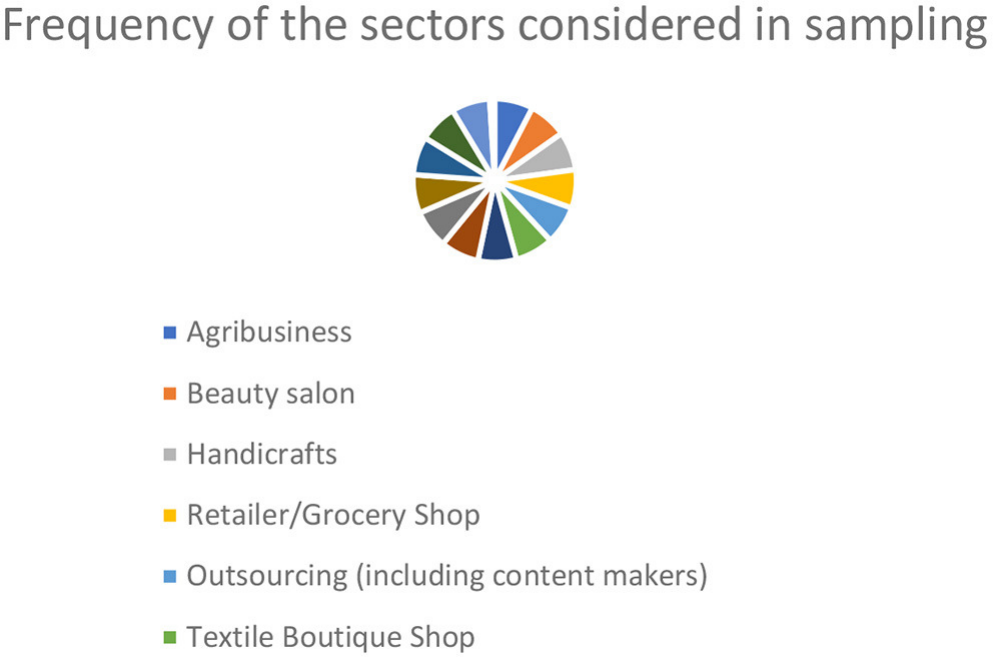
Frequency of the sectors considered in sampling.

**Figure 3:**
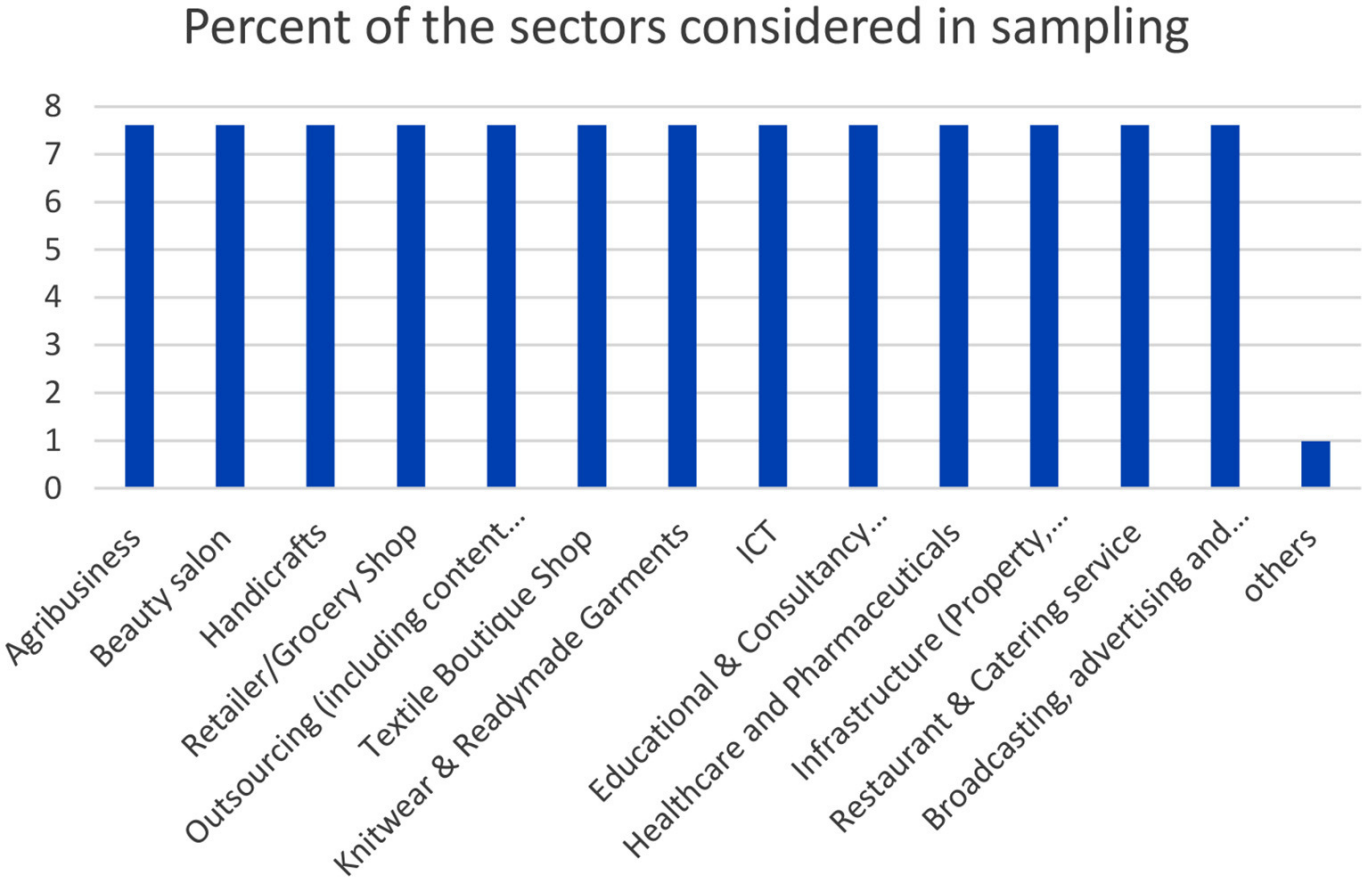
Frequency of the sectors considered in sampling.

The associated graphs related to Industry/Sector Categorisation are given in Figures 2, 3.

The list of variables used in this research is given in Table 2.

**Table 2:** List of variables used in this research.

**Hypothesis**	**Independent variable**	**Dependent variable**	**Moderator**
H1	Perceived ease of use (PEU)	Behavioural intention (BI)/Adoption of OPS	Not applicable (NA)
H2	Perceived usefulness (PU)	Behavioural intention (BI)/Adoption of OPS	Not applicable (NA)
H3	Perceived ease of use (PEU)	Behavioural intention (BI)/Adoption of OPS	Gender
H4	Perceived usefulness (PU)	Behavioural intention (BI)/Adoption of OPS	Gender
H5	Behavioural intention (BI)/Adoption of OPS	Use of OPS	
H6	Use of OPS	SME performance (annual firm revenue)	
H7	Use of OPS	SME performance (annual firm revenue)	Gender

It is mentionable here that the female was coded as 0 and the male as 1 for the measurement of gender (moderator).

Data has been analysed by applying STATA using Bivariate Analysis (Pearson Correlation Coefficient) and Multiple Hierarchical Regression Analyses. This research is divided into two sections. While the first section examines online payment system adoption via the perspective of TAM, the second part uses RBV to investigate the impact of such adoption (use) on performance. Thus, utilising structural equation modelling (for TAM-related hypotheses) and hierarchical regression analysis, the following hypotheses are investigated in the context of SMEs in Bangladesh in order to serve the research's goals (for RBV related hypothesis). Please see Table 3 for more information.

**Table 3:** Theoretical Framework related to hypotheses.

**Hypotheses**	**Theoretical framework**
H1: Entrepreneurs' perceived ease of use (PEU) positively influences their behavioural intention (BI) of adopting the online payment systems in their SMEs.	Explaining adoption with TAM
H2: Entrepreneurs' perceived usefulness (PU) positively influences their behavioural intention (BI) of adopting the online payment systems in their SMEs.	
H3: Gender moderates perceived ease of use (PEU) toward entrepreneurs' adoption of the online payment systems in their SMEs.	
H4: Gender moderates perceived usefulness (PU) toward entrepreneurs' adoption of the online payment systems in their SMEs.	
H5: Entrepreneurs' behavioural intention (BI) of adopting the online payment systems positively influences their use of the online payment systems in their SMEs.	
H6: Entrepreneurs' use of the online payment systems as a strategic resource is positively associated with the financial performance of their organisations.	Examining impact with RBV
H7: Gender of the entrepreneurs moderates the OPS use and SME performance relationship in such a way that the use of OPS adds more to performance in male-led SMEs compared to female-led SMEs.	

## Results

The results of hypotheses testing are provided below (see Table 4).

**Table 4:** Outcomes of hypotheses test.

**Hypothesis**	**Confirmed in …**		**Conclusion for hypothesis**
	**Regression analysis**	**SEM**	
**H1:** Entrepreneurs' perceived ease of use (PEU) positively influences their behavioural intention (BI) of adopting the online payment systems in their SMEs.	Yes (0.315^***^)		Supported
**H2:** Entrepreneurs' perceived usefulness (PU) positively influences their behavioural intention (BI) of adopting the online payment systems in their SMEs.	Yes (0.603^***^)		Supported
**H3:** Gender moderates perceived ease of use (PEU) toward entrepreneurs' adoption of the online payment systems in their SMEs.	Yes (−0.453^**^)		Supported
**H4:** Gender moderates perceived usefulness (PU) toward entrepreneurs' adoption of the online payment systems in their SMEs.	Yes (0.502^***^)		Supported
**H5:** Entrepreneurs' behavioural intention (BI) of adopting the online payment systems positively influences their use of the online payment systems in their SMEs.	Yes (0.227^**^)		Supported
**H6:** Entrepreneurs' use of the online payment systems as a strategic resource is positively associated with the financial performance of their organisations.		Yes (0.596^***^)	Supported
**H7:** Gender of the entrepreneurs moderates the OPS use and SME performance relationship in such a way that the use of OPS adds more to performance in male-led SMEs compared to female-led SMEs.		Yes (0.447^***^)	Supported

*** *p < 0.01,*

** p < 0.05, ^*^p < 0.1.

The above table shows that Gender moderated relationships of PEU and PU to behavioural intention lead to the use of the online payment system (OPS) at the SMEs in Bangladesh (see Table 5). In PEU to BI relation, gender moderates the relationship in favour of females while it does the same for males in PU to BI relationship. The findings indicate that the online payment system adoption model that works in developed nations is also compatible with emerging economies (e.g., Verma and Bhattacharyya, 2017; de Luna et al., 2019).

**Table 5:** Output of the structural model—online payment system adoption in SMEs in Bangladesh.

	**Hypothesis path**	**Path coefficient(β)**	***P*-value**	**Decision**	**Fit indices**
H1	PEU → BI	0.315	0.001^***^	Supported	P-value of Chi square = 0.243; SRMR = 0.017; TLI = 0.971; CFI = 0.993; and RMSEA = 0.031
H2	PU → BI	0.603	0.000^***^	Supported	
H3	Gender^*^PEU → BI	−0.453	0.027^**^	Supported	
H4	Gender^*^PU → BI	0.502	0.004^***^	Supported	
H5	BI → OPS	0.227	0.023^**^	Supported	

*** *p < 0.01,*

** *p < 0.05,*

* *p < 0.1*.

According to the findings of the hierarchical regression, there is a significant positive association between the use of online payment systems and the performance of SMEs. Moreover, hierarchical regression has been used in analysing the moderating impact of gender on OPS use and SME performance relationship (see Table 6).

**Table 6:** Regression outputs—the impact of the online payment system on SME performance.

	**Model 1**	**Model 2**
	**SME performance**	**SME performance**
Location	0.007 (0.011)	0.009 (0.011)
SME industry	0.670^*^ (0.325)	0.723^**^ (0.321)
Entrepreneur's age	0.060^***^ (0.019)	0.056^***^ (0.019)
Start-up capital requirement	0.037^***^ (0.004)	0.037^***^ (0.004)
Number of employees	0.253^**^ (0.090)	0.289^***^ (0.090)
Firm age	0.174^**^ (0.074)	0.104 (0.075)
Type of enterprise (B2B/B2C/B2G)	0.462^***^ (0.135)	0.550^***^ (0.135)
Entrepreneurs' gender	1.221^***^ (0.134)	1.031^***^ (0.146)
**Independent variable**		
Online payment system use		0.596^***^ (0.152)
Gender^*^Online payment system use		0.447^*^ (0.189)
R-squared	0.556	0.670
Constant	10.156^***^ (1.098)	9.346^***^ (1.102)
Observations	302	302

*Standard errors in parentheses;*

*** *p < 0.01,*

** *p < 0.05,*

* *p < 0.1*.

All control and independent variables are significantly correlated with the dependent variable, with the exception of location, and there is no multicollinearity (checked by Pearson correlation coefficient and VIF tests).

## Discussion and Conclusion

### Main Findings

This study was meant to explore the moderating role of gender on the relationship between the online payment system and SME performance in Bangladesh. The research findings support that both Perceived ease of use and Perceived usefulness of OPS has a positive influence on the adoption of OPS in SMEs in Bangladesh. It was also found that the adoption of OPS has a positive influence on the use of OPS in SMEs in Bangladesh. Moreover, it was found that the use of OPS impacts SME performance positively in Bangladesh. Prior research findings (Barkhordari et al., 2017; Mehrad and Mohammadi, 2017; Ameen et al., 2020) matched the findings of this study.

The OPS is increasingly becoming vital for enterprises all over the world (Krotov, 2017). I explored this research model from the Bangladeshi context for two reasons. First, the role of SMEs has been considered one of the main engines of economic growth in an emerging economy like Bangladesh (GOB, 2017). Second, there has been a proliferation of OPS use in the SME sector in Bangladesh in recent days (Neger and Uddin, 2020). However, there have been very few studies exploring OPS in the context of Bangladesh and adopting a gendered approach in doing so.

While it might be argued that the results are not completely counter-intuitive, the context of the research has to be taken into consideration. Very few studies on this topic have been conducted in the context of Bangladesh, which is considered an emerging economy that has just upgraded to a lower-middle-income status recently (World bank, 2021). This is one of the original contributions of this research.

The findings of this study explain the significant association between each independent variable and its associated dependent variable, supporting the TAM framework, as well as TAM's novel contributions to the adoption of e-payment in Bangladesh and the moderating role of Gender on OPS adoption.

One of this study's novel theoretical contributions is to explore whether gender differences can moderate the impact of perceived usefulness and ease of use on the intention to adopt e-payments technology. There have been a number of studies on OPS adoption in SMEs but very few explored the moderating impact of gender on that relationship. Denaputri and Usman (2019), for example, looked at the direct impact of reported ease of use and perceived usefulness on the intention of adopting mobile payment without accounting for gender moderating effects. Roca et al. (2009) also looked at the impact of perceived usefulness and ease of use on e-investor perception in online trading systems, but there were no gender differences in their findings.

The findings of this study on the moderating impact of gender on perceived ease of using OPS show that for female users in SMEs, this factor is more important than male users. With the rise of the use of technologies by women in recent years, one school of thought is that perceived ease of use is not impacted by gender differences. However, this study supports the beliefs of other schools of thought that perceived ease of use is still a determining factor for SMEs in Bangladesh in adopting OPS. This finding is in line with the prior findings by Khan et al. (2020). Women are sometimes uncomfortable utilising internet-based services because they believe they are too difficult. This indicates a lack of self-assurance and the anxiety that comes with it (Chawla and Joshi, 2020). According to Moon and Kim (2001), females will be less at risk from easy-to-use information technology systems. Females are also often reticent to embrace new technologies.

The findings of this study on the moderating impact of gender on the perceived usefulness of using OPS show that for male users in SMEs, this factor is more important than female users. This finding is in line with the prior findings by Schmidthuber et al. (2020). According to both these studies, males are more driven by instrumental motivations than women, and men evaluate perceived usefulness to a higher extent than women when making decisions concerning the usefulness or performance-related components of new technology.

The research findings indicate that OPS not only provides enhanced benefits to organisations but also to consumers. As a result, the OPS is capable of creating a win-win situation for both the enterprises and common people (Saarikko et al., 2020). Hence, this has the potential of creating both social and public value. Hence, the enterprises should consider adopting it. At the same time, the Govt. should make congenial policies to make the adoption of the online payment system easy for enterprises.

According to the resource-based view, H7, which states that the gender of the entrepreneurs moderates the OPS and SME performance relationship in such a way that the application of OPS adds more to performance in male-led SMEs in developing countries than in female-led SMEs, is supported. This disparity could be explained by gender disparities in OPS usage. According to RBV, owner-managers' strategic decisions are influenced by the firm's tangible and intangible resources (Lerner and Almor, 2002; Edelman et al., 2005). Human capital is frequently viewed as the most essential resource at the corporate level, according to Firkin (2001). It has recently been recognised that the demographic characteristics of the entrepreneurs provide personal capabilities (intangible and inimitable assets) that impact performance (Markman and Baron, 2003).

### Contributions and Implications to Research

The findings of this research have manifold contributions to the extant literature. First of all, this research examines the TAM in the context of RBV. Thus, it adds to the technology and ICT literature. By exploring the interplay among the factors related to the adoption of OPS, the use of OPS, and firm performance, this study adds value to RBV literature and hence, also contributes to firm performance literature. Firm performance being a critical construct of strategic entrepreneurship, this paper adds to the strategic entrepreneurship literature. Moreover, Gender and SME being the critical construct of the study, this paper adds to the Gender and SME literature. Despite the fact that the existing literature on OPS is widely documented, most of it is undertaken in the context of large enterprises functioning in relatively developed economies (Dandapani, 2017; Das et al., 2018). In the context of emerging economies like Bangladesh, similar literature on SMEs is still in its infancy. As a result, this study will contribute to the development of a knowledge base and a research agenda focusing on SMEs in emerging or least developed economies. Therefore, it contributes to emerging economy literature.

Gender disparities in ICT user behaviour, skills, interest, and attitude have been documented in the literature (Gorbacheva et al., 2019; Lahtinen, 2019). Males outperform females in sophisticated ICT capabilities, according to some research (Sobieraj and Krämer, 2020). However, there have been very few studies on this in the context of Bangladesh. Therefore, this research is critical in enhancing our understanding of factors that impact the adoption of advanced and new technology like OPS from a gendered angle and a developing country perspective.

Moreover, there is limited evidence in the literature that the entrepreneur's gender has a good or negative impact on the OPS-firm performance relationship. This paper not only explores the impact of OPS on the performance of small businesses but also explores whether the gender of the entrepreneur strengthens or weakens that link or not. It is one of the first studies to look into how an entrepreneur's gender influences an OPS- Business Performance links from a Bangladeshi perspective. As a result, this research adds to the gender literature by expanding not only theoretical but also empirical understanding of gender's moderating function in the OPS and the performance connexion in SMEs. This remains one of the paper's most important contributions.

### Implications for Practise

The findings of this study have numerous practical implications for the owner-managers of SMEs. For example, SMEs' owner-managers can more effectively adopt and use online payment system resources and use them as a means of achieving superior business performance if they realise the impact of OPS on SME performance and the moderating impact of SME entrepreneurs' gender on this relationship. In addition, the findings of this study will assist managers in making a better-informed decision about implementing an OPS into their SMEs' overall strategy.

### Implications for Policy

This study has consequences for policymakers who are deciding on ICT infrastructure policies. The government should encourage the adoption and effective use of OPS because they have a favourable influence on SME performance. To begin, emerging economies should enact legislation to create an atmosphere conducive to the adoption of this ICT instrument. This will provide resource-constrained SMEs with even more reasons to invest in this ICT instrument.

To be more precise, the findings of this study could be used to recommend some improvements and modifications to existing policies and strategies in Bangladesh to boost the usage of OPS (particularly by SMEs) to achieve economic growth. Bangladesh is an emerging economy with a large number of SMEs. Bangladesh's government is devoted to utilising ICT's capacity to aid the country's economic growth and to establishing “Digital Bangladesh.” To this end, Bangladesh's government should revise its ICT policy and continue and enhance measures such as eliminating all taxes on computer hardware, providing interest-free loans to ICT (software) companies, and launching pro-ICT initiatives, grants, incentives, and motivational programmes to encourage the use of ICT in various sectors of the economy.

The development of human resources, particularly women inside SMEs, is a critical component in achieving the goal of a “Digital Bangladesh.” This can be done through lowering training costs, which can be done through a variety of policy instruments. To develop internal knowledge and skills at the firm level, public policies should provide incentives for businesses to invest in their employees' training and specialisation.

### Limitations and Avenues for Future Research

Acknowledging certain limitations, the study suggests numerous directions for future research. First and foremost, the research was quantitative. As a result, future researchers may choose to use qualitative research methodologies to uncover more in-depth discoveries that will add to the body of knowledge.

Though OPS is important to conduct transactions in this pandemic-ridden world, this paper did not relate the implication of the study to the pandemic. This paper looks at the research question in a pre-pandemic period as the data has been collected at that time. OPS in the pandemic ridden world can definitely be the area of research in some future papers.

The data for this study was collected from Bangladeshi SMEs. To obtain more robust research results, future researchers may utilise a cross-country sample technique.

These studies have explored the impact of Perceived ease of use and Perceived usefulness of TAM. However, other elements of the TAM model were not considered in the study. For instance, technology affordability, trust, privacy, and security are associated with technology adoption. The reason for this is this study was conducted in the context of Bangladesh, and a few studies (e.g., Brown, 2002; Kalayou et al., 2020) show that in emerging economies, perceived ease of use and usefulness are more relevant than those other elements of TAM. However, future studies can explore these elements of TAM in emerging economy contexts, as well as these might be relevant after a period of time.

Future studies can also explore the impacts of OPS on firm performance (as well as the moderating effect of gender) in the different business sectors. In this way, more light can be shed on which business sectors the OPS has more positive impacts on firms' performances and which sectors gender plays a more important role.

## Data Availability Statement

The raw data supporting the conclusions of this article will be made available by the authors upon request.

## Ethics Statement

The studies involving human participants were reviewed and approved by Essex University. The patients/participants provided their written informed consent to participate in this study.

## Author Contributions

The author confirms being the sole contributor of this work and has approved it for publication.

## Conflict of Interest

The author declares that the research was conducted in the absence of any commercial or financial relationships that could be construed as a potential conflict of interest.

## Publisher's Note

All claims expressed in this article are solely those of the authors and do not necessarily represent those of their affiliated organizations, or those of the publisher, the editors and the reviewers. Any product that may be evaluated in this article, or claim that may be made by its manufacturer, is not guaranteed or endorsed by the publisher.

## Footnotes

^1^In case the founder is not involved with the SME any longer, the questionnaires were filled out by the owner who possesses the majority ownership of the firm and also actively involved in the firm. In the case of the founders as well, I chose them who are actively involved in the firms.
